# Editorial: From Consumer Experience to Affective Loyalty: Challenges and Prospects in the Psychology of Consumer Behavior 3.0

**DOI:** 10.3389/fpsyg.2017.02224

**Published:** 2017-12-19

**Authors:** María P. Martínez-Ruiz, Mónica Gómez-Suárez, Ana I. Jiménez-Zarco, Alicia Izquierdo-Yusta

**Affiliations:** ^1^Comercializacion e Investigación de Mercados, University of Castilla-La Mancha, Albacete, Spain; ^2^Comercializacion e Investigación de Mercados, Autonoma University of Madrid, Madrid, Spain; ^3^Economic and Business Studies, Open University of Catalunya, Barcelona, Spain; ^4^Comercializcion e Invetigacion de Mercados, University of Burgos, Burgos, Spain

**Keywords:** consumer behavior, consumer emotional journey, market, marketing 3.0, marketing 4.0

Around the world, consumer markets have been fundamentally changed by various forces, such as globalization and social media. As a result, organizational managers are increasingly challenged to analyze and understand consumer behavior. Indeed, companies' long-term competitiveness may depend on how well they understand diverse product markets and can adapt to consumers' needs and demands.

This research topic for Frontiers in Psychology highlights some of the more relevant changes that have conditioned consumer behavior in recent years—among these, the paradigm shift in marketing is worth emphasizing.

Indeed, when the call for papers began in September 2015, marketing studies have already recognized the transition from marketing 1.0 to marketing 3.0. For instance, Kotler et al. (2010) suggest how along time marketing orientation within organizations passed through three different stages: marketing 1.0 oriented to the product, marketing 2.0 focused on the client (2.0) and from there to marketing 3.0, “the Values Driven Era,” whereby consumers' personal values (ethical, social, spiritual, etc.) were incorporated into marketing decisions. Whilst under traditional marketing unidirectional communication was the key, currently connectivity and technology have altered the way marketing approach to consumers.

By the end of 2016, the Marketing 4.0 perspective has emerged. Its goal is to help organizations reach and engage consumers more fully than in previous years by analyzing shifts in consumers' behaviors (Kotler et al., 2017). Thus, Marketing 4.0. represents the natural evolution of Marketing 3.0., based on the use of technologies as a way to know, dialogue, interact, and establish a relationship with the consumers (Jiménez-Zarco et al., 2017). Moreover, Marketing 4.0 emphasizes the need to consider simultaneously the “new” and the “old” marketing to get consumers recommend the brand. Especially through the numerous possibilities social media and digital marketing offer in this respect, which are revolutionizing the marketing environment and consequently, the way business are being made today.

Today, the market—considering in a wide sense all stakeholders and not only end consumers—is forcing companies to implement Marketing 4.0; but also, companies push the market towards Marketing 4.0, so it is possible to affirm this phenomenon is taking place in a double direction. However, not all agents are adapting at the same time to the use of new technologies.

This new marketing approach modifies both the business rules and the channels by changing the relationship with consumers. Nowadays, clients appreciate constant communication with the organization and expect more than a relationship based on reciprocity and interactivity; they expect the company to “contribute with something else” (Gómez-Suárez et al., 2016), especially in the realm of emotional value (Kotler et al., 2017).

In particular, there is a transformation of the relation between the company and the client, as well as a modification of the playing rules and the relationship channels between both agents (Melero et al., 2016). For this reason, organizations must continue to offer value to the client, according to the digital environment (Vernuccio et al., 2015) and taking into account an inclusive marketing of attraction, oriented to create valuable contents, and offer satisfactory experiences, with the aim at increasing affective links with brands (Kaufmann et al., 2016).

While those facets of the new consumer paradigm are approached, there remain pivotal questions about consumer behavior. To address these gaps, the present Research Topic brings together 30 studies by 76 authors who analyzed the relevance of consumer behavior changes using different theoretical and methodological frameworks. These different papers, mainly constituting original research, examine a variety of sub-topics, including online and mobile environments, value co-creation, internal marketing strategies, and diverse industries and product markets. In particular, these research gaps can be described as follows:

- Which are the features that best distinguish the adaptation of consumers segments to the development of new technologies?
- Could it be suggested that consumers are responding to companies' co-creation proposals, thus fostering the evolution of Marketing?
- Is it possible to apply the same way Marketing 4.0 to all industries and sectors? If not, which are the most permeable ones to Marketing 4.0?
- Is internal marketing being used as part of the strategies companies use to evolve from Marketing 3.0 to Marketing 4.0? If so, how is it being applied?

In the last decade, the advance of the Internet and other new technologies has transformed both markets and consumers behaviors. Seven articles focusing on online and mobile environments highlight instances of companies adapting the following new strategies and tools. Omni channel (Juaneda-Ayensa et al.), mobile advertising (Hongyang and Zankui; Martínez-Ruiz et al.), virtual reality (Charron), promotional and online services through web pages (Bláquez-Resino et al.; Méndez-Aparicio et al.), and electronic word of mouth (Huete-Álcocer).

Another four articles discuss the value co-creation—whereby many companies and organizations are giving consumers a more active role in the value-creation process. Through their literature review, Gómez-Suárez et al. examine how consumer-brand relationships have received increasing attention in recent years. Martínez-Cañas et al. highlight how those brands acknowledged as ethical elicit positive emotional responses among their consumers. Houdek shows that even socially and environmentally responsible consumers (the so-called Consumer 3.0) exhibit selective recall, limited attention, and bounded search in their perceptions about the price-quality of purchases. Ruiz de Maya et al. analyze how companies' allocation of resources to corporate social responsibility (CSR) initiatives serve as a form of value co-creation with consumers.

Several articles address issues related to diverse industries and sectors. Four papers emphasize *health* (Múñoz et al.; Pelegrín-Borondo et al.; Selva-Sevilla et al.; Selva-Selvilla et al). Three studies focus on *retailing* (Garrido-Morgado et al.; Medrano et al.; Villacé-Molinero et al.). Three concentrate on *banking and financial* markets (Callejas-Albiñana et al.; Cano et al.; González et al.). There are another two studies about *food* (Hidalgo-Baz et al.; Martínez-Ruiz and Gómez-Cantó), two about *non-profit organizations* (Callejas-Albiñana et al.; Juaneda-Ayensa et al.), and one about *transport* (Muro-Rodríguez et al.).

Finally, internal marketing strategies are another important part of the new paradigm in consumer research. On this topic, three papers explain how companies are adopting new organizational strategies in order to appeal to customers in the current millennium. Linuesa-Langreo et al. explain the impact of servant leadership, which involves putting employees' needs first and serving the broader society. Gutiérrez-Boncano et al. determine the relevant aspects of family businesses that make them increasingly competitive. González and Fernández focus on the inclusion of people with disabilities, while Torrent-Sellens et al. focus on dispositional employability.

Given this broad selection of papers, we encourage readers to draw their own conclusions about the complex phenomena of consumer behavior. Our hope is that these different perspectives will cover various gaps in the field and prompt discussion among the audience of Frontiers in Psychology.

## Author contributions

All authors listed have made a substantial, direct and intellectual contribution to the work, and approved it for publication.

### Conflict of interest statement

The authors declare that the research was conducted in the absence of any commercial or financial relationships that could be construed as a potential conflict of interest.

## References

[B1] Gómez-Suárez M. Alonso L. Campo S. (2016). Exploring the link between brand love and engagement through a qualitative approach. Int. J. Bus. Environ. 8, 367–384. 10.1504/IJBE.2016.10001655

[B2] Jiménez-Zarco A. I. Rospigliosi A. Martínez-Ruiz M. P. Izquierdo-Yusta A. (2017). Marketing 4.0: Enhancing Consumer-Brand Engagement through Big Data Analysis, in Socio-Economic Perspectives on Consumer Engagement and Buying Behavior. Hershey, PA: IGI Global, 94–117.

[B3] Kaufmann H. R. Correia Loureiro S. M. Manarioti A. (2016). Exploring behavioural branding, brand love and brand co-creation. J. Prod. Brand Manage. 25, 516–526. 10.1108/JPBM-06-2015-0919

[B4] Kotler P. Kartajaya H. Setiawan I. (2010). Marketing 3.0: From Products to Customers to the Human Spirit. Hoboken, NJ: John Wiley and Sons.

[B5] Kotler P. Kartajaya H. Setiawan I. (2017). Marketing 4.0: Moving from Traditional to Digital. Hoboken, NJ: John Wiley and Sons.

[B6] Melero I. Sese F. Verhoef P. C. (2016). Recasting the customer experience in today's omni-channel environment. Univ. Bus. Rev. 2016, 18–37. 10.3232/UBR.2016.V13.N2.01

[B7] Vernuccio M. Pagani M. Barbarossa C. Pastore A. (2015). Antecedents of brand love in online network-based communities. A social identity perspective. J. Prod. Brand Manage. 24, 706–719. 10.1108/JPBM-12-2014-0772

